# Multimodal fusion with deep neural networks for leveraging CT imaging and electronic health record: a case-study in pulmonary embolism detection

**DOI:** 10.1038/s41598-020-78888-w

**Published:** 2020-12-17

**Authors:** Shih-Cheng Huang, Anuj Pareek, Roham Zamanian, Imon Banerjee, Matthew P. Lungren

**Affiliations:** 1 Department of Biomedical Data Science, Stanford University, Stanford, USA; 2 Center for Artificial Intelligence in Medicine and Imaging, Stanford University, Stanford, USA; 3 Department of Radiology, Stanford University, Stanford, USA; 4 Department of Pulmonary Critical Care Medicine, Stanford University, Stanford, USA; 5 Vera Moulton Wall Center for Pulmonary Vascular Disease, Stanford University School of Medicine, Stanford University, Stanford, USA; 6 Department of Biomedical Informatics, Emory University, Atlanta, USA

**Keywords:** Embolism, Data integration, Machine learning

## Abstract

Recent advancements in deep learning have led to a resurgence of medical imaging and Electronic Medical Record (EMR) models for a variety of applications, including clinical decision support, automated workflow triage, clinical prediction and more. However, very few models have been developed to integrate both clinical and imaging data, despite that in routine practice clinicians rely on EMR to provide context in medical imaging interpretation. In this study, we developed and compared different multimodal fusion model architectures that are capable of utilizing both pixel data from volumetric Computed Tomography Pulmonary Angiography scans and clinical patient data from the EMR to automatically classify Pulmonary Embolism (PE) cases. The best performing multimodality model is a late fusion model that achieves an AUROC of 0.947 [95% CI: 0.946–0.948] on the entire held-out test set, outperforming imaging-only and EMR-only single modality models.

## Introduction

Pulmonary Embolism (PE) is a serious medical condition that hospitalizes 300,000 people in the United States every year^1^. The gold standard diagnostic modality for PE is Computed Tomography Pulmonary Angiography^2^ (CTPA) which is interpreted by radiologists. Studies have shown that prompt diagnosis and treatment can greatly reduce morbidity and mortality^3^. Over the past 20 years, the usage of CTPA in the emergency department alone has increased 27-fold^4^, and still, patients with PE experience more than 6 days of delay in diagnosis and 26% of patients are misdiagnosed during their first visit^5,6^. Strategies to automate accurate interpretation and timely reporting of CTPA examinations may successfully triage urgent cases of PE to the immediate attention of physicians, improving time to diagnosis and treatment.

Many studies have reported promising results in applying deep learning models to automate diagnosis in medical imaging^7–10^, including PE diagnosis on CTPA^11–13^. While prior work has demonstrated potential for accurate automated image analysis based on imaging data alone, this is in contrast to routine clinical practice in which medical imaging is interpreted along with relevant clinical data to inform accurate diagnosis. In fact, clinical data availability during image interpretation is particularly important in radiology, as accurate medical diagnosis on imaging often relies significantly on pre-test probability, prior diagnosis, clinical and laboratory data, and prior imaging. For example, a survey showed that more than 85% of radiologists consider clinical context as vital for radiological exam interpretation^14^. This also holds true in the use case of pulmonary embolism diagnosis on CTPA where clinical context and prior imaging results are considered important for imaging decisions.

Multimodal data fusion for automated clinical outcome prediction and diagnosis has been gaining traction within the past 3 years. For prediction of Alzheimer's disease^15–18^, demographic data with specific lab tests were combined with imaging data as inputs to deep learning models and found improvement over single data source models. Similarly, combining patient demographic information with dermatoscopic images of skin lesions observed a boost in performance as compared to single modality skin cancer models^19–21^. Other studies have seen similar advantages in a diverse set of medical imaging tasks such as breast cancer prediction, glaucoma classification and detection of microcytic hypochromia^22–24^. Yet, despite the promise of multimodal fusion techniques, prior work has focused on approaches using only one of several possible fusion techniques and relying on just a few manually selected clinical features. Understanding how leveraging more feature-rich clinical datasets for multimodal fusion can impact model performance and the relative performance of different fusion techniques have not yet been explored.

**Figure 1:**
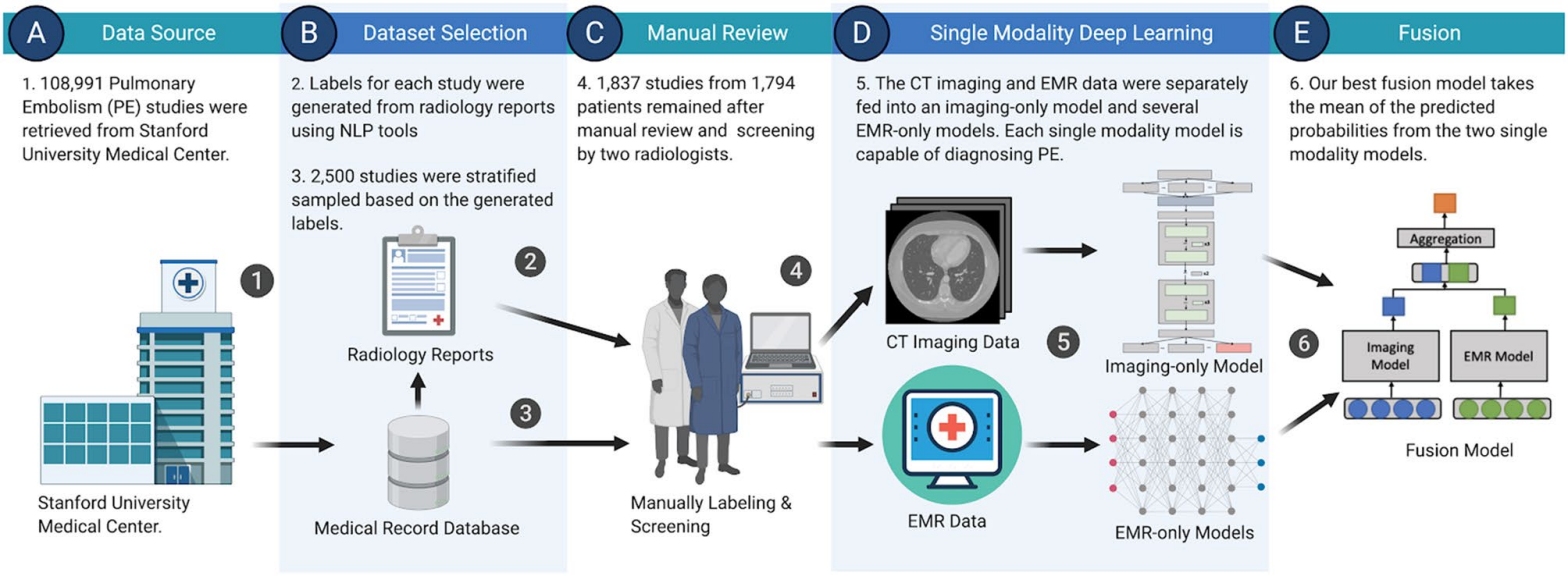
Overview of the workflow for this study. We extracted a total of 108,991 studies from Stanford University Medical Center (**A**) and sampled a subset (**B**) for manual review (**C**). 1837 studies remained after screening by two radiologists and were used to train and evaluate our models. Single modality models were created (**D**) both as baselines for comparisons as well as components for the fusion models (**E**).

The purpose of this study is to build and compare multimodal fusion models that combine information from both CT scans and Electronic Medical Record (EMR) to automatically diagnose the presence of PE. Leveraging both clinical and imaging data by using a variety of fusion approaches could not only lead to a contextually relevant model which reduces PE misdiagnosis rate and delay in diagnosis, but also inform future work by exploring optimal data selection and fusion strategies. Figure 1 outlines the overall workflow used for this study.

## Results

**Figure 2:**
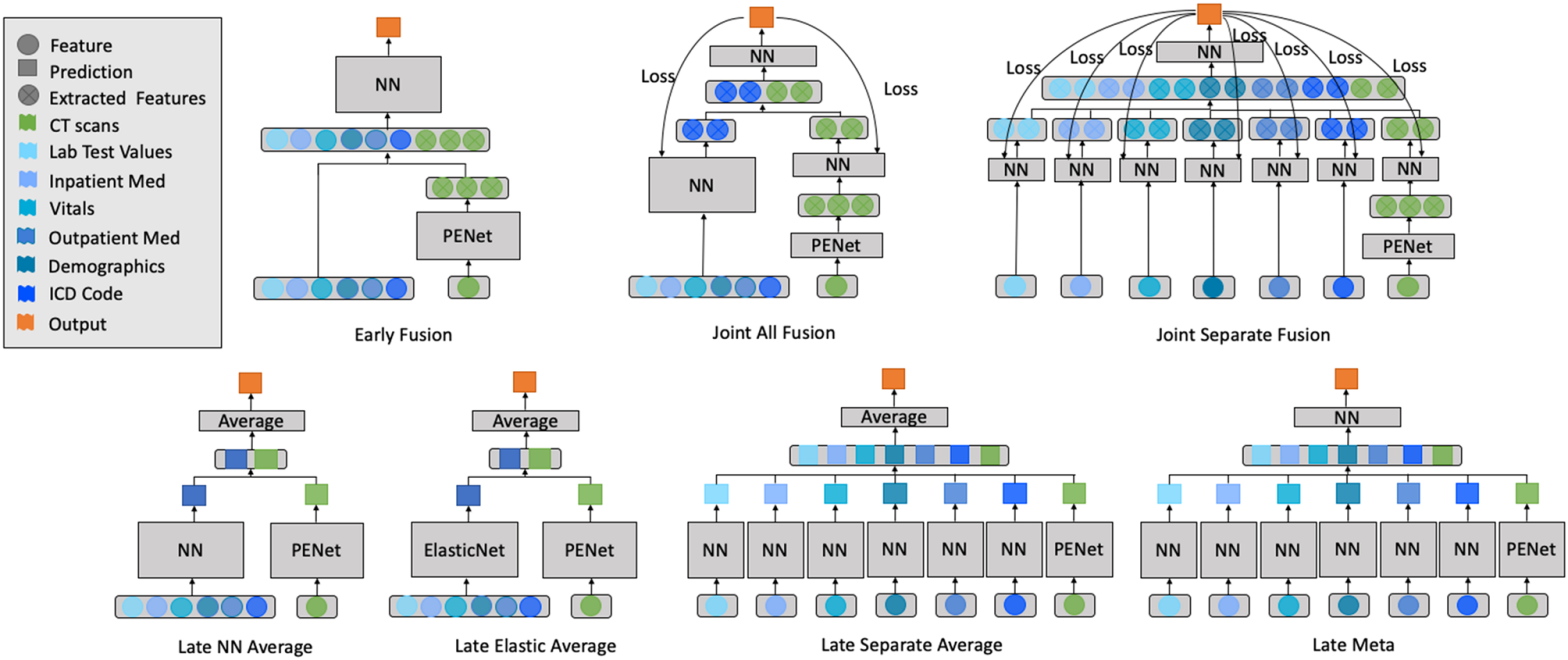
Fusion model architectures. The 7 different fusion architectures used in this study, including (**A**) Early Fusion, (**B**) Joint All Fusion, (**C**) Joint Separate Fusion, (**D**) Late NN Average, (**E**) Late Elastic Average, (**F**) Late Separate Average and (**G**) Late Meta. Each input feature modality is color coded. Detailed definition of each model architecture is described in the Methods.

In this study, we separately trained an imaging-only model (PENet)^12^ and 7 EMR-only neural network models (details in Methods). These single modality models not only serve as baselines for performance comparison, they also provide different inputs and components for different fusion models. A total of 7 fusion architectures were implemented and compared (Fig. 2).

### Data acquisition

With the approval of Stanford Institutional Review Board (IRB), we retrieved 108,991 studies from patients that had CTPA performed under the pulmonary embolism protocol between 2000 and 2016 at Stanford University Medical Center (SUMC). To curate a labeled dataset for training and testing with equal distribution of positive and negative PE cases, 2500 studies were selected by stratified random sampling from the original 108,991 studies, based on Natural Language Processing generated labels from radiology reports^25,26^. After removing studies with wrong protocols, significant artifacts, poor imaging quality and nondiagnostic studies, 1837 studies from 1794 patients out of the 2500 sampled subset remained.

For each study that remained after screening, axial CT imaging data with slice thickness of 1.25 mm were pulled from the local picture archiving and communicating system servers. The CT scans and radiology reports for each study were separately reviewed by two board certified radiologists to create ground truth labels of PE diagnosis. The standard descriptions of central positive, segmental positive, subsegmental positive and negative PE by Remy-Jardin et al.^27^ was used for labeling ground truth. Slight modifications to the descriptions were made to account for anatomic variations and the orientation of vessels in the transverse plane on the CT scans. Particularly, subsegmental-only PE was defined as the location of the largest defect at the subsegmental level on a spiral CT, allowing a satisfactory visualization of all pulmonary arteries at the segmental level or higher. Furthermore, slice-wise labels of positive PEs were made on all of the PE positive cases. The two radiologists had a high inter-rater reliability (Cohen’s Kappa Score of 0.959) and the senior radiologist resolved all conflicted cases.

For each of these studies, we also pulled a comprehensive view of patient EMR from the SUMC Epic database within an observational window of 12 months prior to their CT examination date. The EMR includes ICD9 codes, vitals, lab tests, demographics and inpatient and outpatient medications.

We randomly split the studies into a training set (1454 studies from 1414 patients), a validation set (193 studies from 190 patients) and a hold-out test set (190 studies from 190 patients) for the purpose of developing the models. We ensured that there was no patient overlap between each set. The detailed characteristics of our dataset can be found in Table 1.

**Table 1:** Data characteristics of the Stanford Medical Center dataset.

Category	Sub-category	Overall	Train	Validation	Test
CTPA exams	Number of studies	1837	1454	193	190
	Number of patients	1794	1414	190	190
	Median number of slices (IQR)	386 (134)	385 (136)	388 (132)	388 (139)
Patient Demographics	Female	1048 (57.07%)	823 (56.64%)	130 (67.36%)	99 (52.08%)
	Median age (IQR)	66.14 (53.24–82.40)	66.13 (53.14–82.95)	64.10 (50.88–78.38)	67.24 (56.62–82.76)
Race	White	1101 (59.70%)	872 (59.80%)	108 (55.96%)	121 (62.69%)
	Black	140 (7.59%)	101 (6.93%)	22 (11.40%)	17 (8.80%)
	Asian	144 (7.81%)	122 (8.37%)	12 (6.21%)	10 (5.18%)
	Pacific Islander	13 (0.70%)	10 (0.69%)	0 (0.0%)	3 (1.55%)
	Other	210 (11.39%)	168 (11.52%)	25 (12.95%)	17 (8.80%)
	Unknown	233 (12.63%)	184 (12.62%)	24 (12.44%)	25 (12.95%)
Pulmonary embolism	Number of negative PE	1111(60.48%)	946 (65.06%)	85 (44.04%)	80 (42.10%)
	Number of positive PE	726 (39.50%)	508 (34.94%)	108 (55.96%)	110 (57.89%)
	Central	257(35.40%)	202 (39.76%)	27 (25.00%)	28 (25.45%)
	Segmental	387(53.31%)	281 (55.31%)	52 (48.15%)	54 (49.09%)
	Subsegmental	82 (11.29%)	25 (4.91%)	29 (26.85%)	28 (25.45%)
Vitals	BMI (mean: std)	28.37 : 9.65	28.36 : 10.03	27.11 : 6.78	29.60 : 9.22
	Pulse (mean: std)	81.62 : 14.99	81.53 : 15.64	83.05 : 11.86	80.50 : 13.06
D-dimer	D-dimer test taken	580 (30.62%)	461 (30.90%)	58 (28.71%)	61 (30.50%)
	D-dimer positive	496 (26.18%)	389 (26.07%)	51 (25.25%)	56 (28.00%)

The curated Stanford Medical Center dataset was divided into training, validation and test set. The training set was used to optimize model parameters and the validation set was used to select the best model hyperparameters and operating points. The hold-out test set was used to evaluate the model’s performance.

### Model performances

Each feed-forward neural network (including EMR-only and fusion) was trained using a grid search approach to find the optimal hyperparameters. The best hyperparameters, along with the training and validation metrics, of all of the grid searched models are detailed in Supplementary Table S1. All of the models achieved their lowest validation loss before the last iteration, which implies that the saved models have converged before the last epoch. The **Late Separate Average** and **Late Meta** fusion models are built based on the 7 best single modalities models with these grid search hyperparameters. The performance of each fusion model, including subsegmental PE, is detailed in Table 2. Over the entire hold-out test set, the **Late Elastic Average** model achieved the highest test AUROC of 0.947. Using bootstrap to compute the p-values between each model, we show that late elastic average’s performance outperformed the other fusion architectures significantly (Supplementary Figure S3).

**Table 2:** Fusion model architecture experimentation.

Evaluation metrics	Early	Late				Joint	
	Early fusion	Late NN average	Late elastic average	Late separate average	Late meta	Joint all	Joint separate
Operating threshold	0.345	0.473	0.414	0.483	0.197	0.500	0.517
Accuracy	0.842 [0.84–0.844]	0.848 [0.846–0.849]	**0.885** [0.884–0.886]	0.853 [0.851–0.854]	0.828 [0.826–0.829]	0.809 [0.808–0.811]	0.842 [0.841–0.844]
AUROC	0.899 [0.898–0.901]	0.895 [0.894–0.897]	**0.947** [0.946–0.948]	0.908 [0.906–0.909]	0.896 [0.895–0.898]	0.796 [0.794–0.798]	0.893 [0.891–0.894]
Specificity	0.737 [0.733–0.74]	0.838 [0.835–0.84]	**0.902** [0.9–0.904]	0.851 [0.849–0.853]	0.852 [0.849–0.854]	0.709 [0.706–0.712]	0.837 [0.835–0.840]
Sensitivity	**0.919** [0.918–0.921]	0.781 [0.778–0.783]	0.873 [0.871–0.875]	0.854 [0.851–0.856]	0.81 [0.808–0.813]	0.882 [0.88–0.884]	0.846 [0.844–0.849]
PPV	0.827 [0.825–0.829]	0.869 [0.867–0.871]	**0.924** [0.923–0.926	0.887 [0.886–0.889]	0.883 [0.881–0.885]	0.807 [0.805–0.809]	0.877 [0.875–0.879]
NPV	**0.870** [0.867–0.873]	0.734 [0.731–0.737]	0.838 [0.835–0.84]	0.809 [0.806–0.811]	0.765 [0.762–0.768]	0.814 [0.811–0.817]	0.799 [0.796–0.801]

Comparison between different fusion strategies. Model performance on the held-out test set with 95% confidence interval using probability threshold that maximizes both sensitivity and specificity on the validation dataset. Best performance metrics in bold text.

As detailed in Table 3 and Supplementary Figure S2, our best fusion model significantly outperforms both of our best performing single modality models: 0.036 AUROC higher than EMR only-model and 0.156 AUROC higher than imaging-only model across the entire test set (Supplementary Figure S3). In this study, we set our operating point based on the Youden’s J-Score statistic that maximizes both sensitivity and specificity on the validation set. We used the standard definition of operating point as the numeric threshold that separates the predicted classes: all studies with prediction probability higher than this operating point are considered positive predictions, otherwise negative^28^. Using this threshold, our fusion model achieves better performance across all evaluation metrics as compared to either single modality modals. Applications in clinical settings, however, are usually tuned to maximize sensitivity in order to minimize the false-negative rate. We can further improve the fusion model’s sensitivity with the cost of lowering PPV: using an operating point of 0.35, the fusion model achieves a sensitivity of 0.972 [0.971–0.973] and PPV of 0.862 [0.861–0.864] across the entire test set, as well as sensitivity of 1.00 [1.00–1.00] and PPV of 0.829 [0.827–0.832] when excluding subsegmental cases in the test set.

**Table 3:** Comparison between multimodality and the best performing single modality models.

Evaluation metrics	Including subsegmental			Excluding subsegmental		
	Imaging model	EMR model	Late elastic average	Imaging model	EMR model	Late elastic average
Operating threshold	0.625	0.630	0.448	0.625	0.612	0.414
Accuracy	0.687 [0.685–0.689]	0.834 [0.832–0.835]	**0.885** [0.884–0.886]	0.756 [0.754–0.758]	0.873 [0.871–0.874]	**0.902** [0.900–0.903]
AUROC	0.791 [0.788–0.793]	0.911 [0.910–0.913]	**0.947** [0.946–0.948]	0.833 [0.830–0.835]	0.921 [0.919–0.923]	**0.962** [0.961–0.963]
Specificity	0.862 [0.860–0.865]	0.875 [0.872–0.877]	**0.902** [0.9–0.904]	0.863 [0.861–0.866]	**0.878** [0.876–0.880]	0.849 [0.847–0.852]
Sensitivity	0.559 [0.557–0.562]	0.804 [0.801–0.806]	**0.873** [0.871–0.875]	0.651 [0.647–0.654]	0.867 [0.865–0.870]	**0.953** [0.951–0.954]
PPV	0.848 [0.846–0.851]	0.898 [0.896–0.899]	**0.924** [0.923–0.926	0.830 [0.827–0.833]	**0.879** [0.877–0.882]	0.866 [0.864–0.869]
NPV	0.588 [0.585–0.590]	0.765 [0.761–0.767]	**0.838** [0.835–0.84]	0.707 [0.705–0.710]	0.866 [0.864–0.868]	**0.946** [0.945–0.948]

Model performance on the held-out testset with 95% confidence interval using probability threshold that maximizes both sensitivity and specificity on the validation dataset. Best performance metrics in bold text.

**Figure 3:**
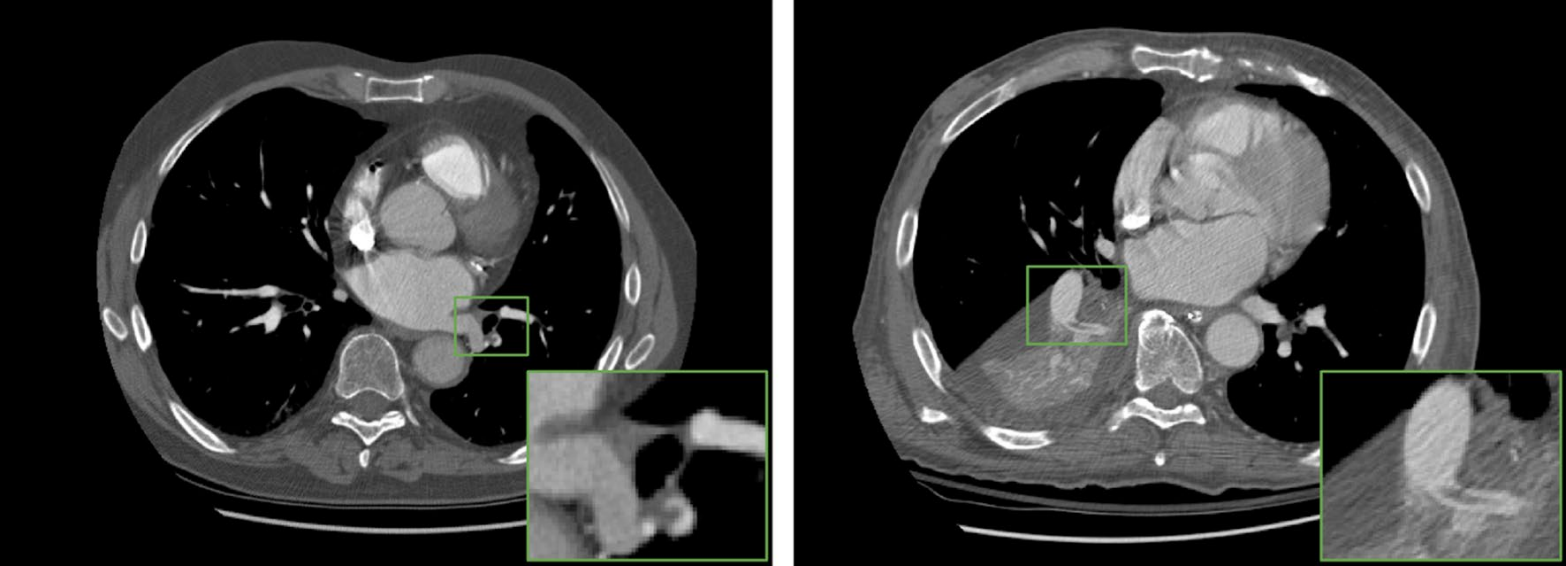
Two selected axial CT images of the chest from two separate patients with positive diagnosis of PE. The left CT scan demonstrates a left lower lobe posterolateral basal segmental artery filling defect consistent with a pulmonary embolism. The CT scan on the right panel demonstrates a small elongated filling defect bridging across the segmental arteries of the right lower lobe consistent with a segmental pulmonary embolism, in addition to surrounding collapse of the right lower lobe. The vision-only model yielded false-negative predictions for both cases, but the fusion model correctly predicted both as positive.

Our qualitative analysis of false-positive predictions (Supplementary Table S4) indicated that all of these studies had pre-existing or concurrent medical conditions. A qualitative analysis of the false-negative predictions made by the vision model showed that these PEs were either (1) subsegmental or very small and difficult to assess even for the radiologist or (2) surrounded by pathological findings such as collapse of lung tissue or pleural effusion (examples in Fig. 3). In 39 out of 49 cases (79.59%), the fusion model was able to correctly re-classify false-negative mistakes made by the vision-only model. Half of the remaining false-negative cases were clinically insignificant subsegmental only PEs. Lastly, our fusion model uses a late fusion approach, which takes the average of two independent models, each using a different modality. In the situation where one of the modalities isn’t present (e.g., before the patient is able to obtain CT imaging), our fusion model is still capable of making predictions based only on the patient’s EMR and could be used for imaging workflow triage.

## Discussion

The purpose of this study was to build a multimodal deep learning model that leverages information from both CT images and Electronic Medical Record (EMR) to diagnose pulmonary embolism. We found that the fusion model achieved state-of-the-art AUROC of 0.962 [0.961–0.963] for detecting clinically important central and segmental PE which was significantly better than either the pixel-based (0.833 [0.830–0.835]) or EMR-based (0.921 [0.919–0.923]) models alone. Setting an operating threshold of 0.35 also allowed our model to capture all positive cases while maintaining PPV of 0.829, supporting potential clinical utility as a screening and prioritization tool to improve time to diagnosis and treatment.

Implementation of neural network models for PE diagnosis started as early as the 1990s^29^. These studies initially focused on using simple neural networks trained with hand-crafted clinical variables or planar ventilation-perfusion imaging with modest performance^30^. More recently, researchers have shifted their attention to applying Convolutional Neural Networks (CNNs) on volumetric CTPA imaging for PE diagnosis. However, prior work relied on extensive feature engineering and processing, frustrating efforts towards clinical deployment given a lack of an end-to-end solution. For example, Tajbakhsh et al.’s 3D CNN requires extensive segmentation and vessel-alignment to extract pixel features as inputs^13^. Similarly, Yang et al.’s 3D CNN also relies on detecting candidate voxels in the entire CTPA volume using a region proposal network as inputs to the classification model^11^. State-of-the-art by Rajan et al. employs a two-stage model capable of achieving 0.85 AUROC for detecting saddle and central PE, and 0.70 AUROC in detecting segmental and smaller PE^31^. In contrast, our work not only relies on an end-to-end solution that avoids complex image pre-processing, but also utilized important clinical and laboratory data with imaging data to achieve a combined AUROC of 0.947 for the task of automatically detecting PE.

Medical imaging diagnosis relies heavily on synthesis of clinical data from multiple sources in order to inform accurate interpretation of the imaging data since substantial clinical context is often essential to drive diagnosis. For example, many prior studies have found that a lack of access to clinical and laboratory data during image interpretation results in lower imaging interpretation performance and decreases clinical utility for the referring provider^32^. The importance of clinical context during image interpretation for clinical decision-making also holds true in the use case of PE diagnosis on CTPA. Recognizing this, we developed multimodal deep learning models for detecting PE using both CT imaging and large-scale patient EMR-data and found that multimodal fusion models significantly outperformed single modality models. In particular, we found that the single modality image-based model showed large overlapping regions of predicted probabilities for the positive and negative test cases, which precludes a clinically useful operating threshold (see Supplementary Figure S5). The EMR only model revealed more defined clusters of the same cases but still suffered from limited separation. To achieve a clinically useful high sensitivity performance, the EMR only model would require a low operating point (0.05) to correctly detect all PE positive cases but would lead to too many false-positives (0.337 specificity). In contrast, the multimodal fusion model achieves more clinically useful separation between positive and negative cases; all the central and segmental positive cases can be diagnosed correctly with an operating point of 0.35 and achieving a specificity 0.778. Based on this analysis the fusion model may be more optimal for integration into clinical workflow due to the end-to-end approach and high sensitivity using a low operating point, thereby helping to reduce false-positives and clinical alert fatigue.

This study includes several important limitations. This is a retrospective study design which comes with well described shortcomings and inherent limitations. The deep learning model described was developed and trained on data from a single large academic institution. Validation on an external test set from another institution has to be done to better understand the generalizability of our model and will inform the direction of future work. Although pre-existing or concurrent medical conditions exist in all of the false-positive cases in the test set, our model is not trained to identify these cases and should not be used explicitly to identify other important pathologies. Our joint fusion models are based on extracted features from the vision model instead of using the original CT scans, which can limit the models’ ability to generate feature representations that might best complement the EMR features. Lastly, our comparison of different fusion types is based only on the task of predicting PE using CT scans and EMR, so the methods and results should be considered with caution when applying to other predictive tasks using different input modalities.

To summarize, the core contributions of this work include development and evaluation of different end-to-end multimodal deep learning models for detecting PE using both CT imaging and patient EMR data. Our best performing model is a late fusion model using 3D CNN and ElasticNet which achieved an AUROC of 0.962 [0.961–0.963]. End-to-end machine learning models that consider both CT scans and patient medical history, similar to a radiologist, offer better abnormality discrimination over imaging data alone. Multimodal data fusion models may improve the clinical utility of automating medical imaging tasks and are well-suited for adoption in clinical practice.

## Methods

### CT imaging only model

In order to observe the effect of using different multimodality fusion strategies, we created single modality classification models as baselines for comparison. To preprocess data for the CT imaging-only model, all of the pixel data for each CT exam were extracted from the original Digital Imaging and Communications in Medicine (DICOM) format. The CT scans were scaled to 224 × 224 × N pixels where N is the number of CT slices. The Hounsfield Units were clipped to the range of − 1000 to 900 and normalized to be zero-centered. During training, a sliding window of 24 slices was fed into the model instead of the entire volumetric CT scan to increase the proportion of the target PE relative to the input. A sliding window was considered PE positive if more than 35% of the slices were labeled as positive.

In our previous work, we have developed a 77-layer 3D Convolutional Neural Network (CNN) model, PENet, capable of detecting PE using only CT imaging^12^. PENet is primarily made up of layers of 3D convolutions with skip connections and squeeze-and-excitation blocks. Some notable implementation details of PENet include (1) pretraining the model with a video dataset (Kinetics-600) for transfer learning and (2) using a sliding window of CT slices as inputs and base prediction on the sliding window with the highest PE probability. The detailed model architecture and training procedure can be found in the original manuscript. Due to its high performance in detecting PE as compared to other classical 3D CNN architectures, we have decided to input CTPA exams to PENet for this study as our imaging only model. After pretraining the model on the Kinetics-600 dataset, we replaced the softmax output layer with a single output neuron with sigmoid activation and continued training with the CT scans from the training dataset. We used a focal loss function^33^ to alleviate the class imbalance between the sliding windows.

### EMR only model

Each category of EMR was parsed and feature engineered in different ways in accordance to the processing steps described by Banerjee et al.^34^. The demographic features consisted of one-hot encoded gender, race and smoking habits and the age as a numeric variable. For vitals, we included systolic and diastolic blood pressure, height, weight, body mass index (BMI), temperature, respiration rate, pulse oximetry (spO2) and heart rate. The vitals were represented with respect to their sensitivity to change, which was computed by taking the derivative of the vital values along the temporal axis. 641 unique classes of drugs were identified for inpatient and outpatient medication. Each medication was represented as both the frequency within the 12-month window and a binary label of whether the drug was prescribed to the patient. We excluded all ICD-9 codes with less than 1% occurrences in the training dataset and collapsed into top diagnosis categories, which resulted in a total of 141 diagnosis groups. We used a binary presence/absence as well as a frequency to represent diagnosis code as features. All ICD codes recorded with the same encounter number as the patient’s CT exam, or within a 24 hour window prior to their CT examination, were dropped to avoid data leakage. Lastly, we identified 22 lab tests and represented each test as binary presence/absence as well as the latest value of the test.

We have implemented a simple feed-forward neural network that uses a concatenation of all EMR features as inputs (except CT imaging features). We hypothesized that the sparse EMR feature vectors would be challenging for neural network models to learn, so we also applied ElasticNet^35^ to detect PE using all the EMR features. As part of the implementation step for the late fusion models, we also implemented feed-forward neural networks for each individual category of EMR features (demographics, ICD-9 codes, vitals, medications, lab tests). Before feeding into each model, all input features are normalized by subtracting the mean and dividing by the standard deviation.

### Fusion models

The processed data used for ‘Imaging only model’ and ‘EMR only model’ was also used for our fusion models. We implemented different fusion architectures that leveraged information from both CT scans and patient EMR using different strategies, namely early fusion, late fusion and joint fusion. Early fusion is defined as joining features or feature representations at the input level before feeding into a model. Late fusion, also known as decision level fusion, aggregates the prediction probabilities of different single modality models to make a final prediction. Joint fusion extracts feature representations from each modality using a neural network model, then concatenates these learned feature representations as inputs to another model. The prediction loss from the fusion model is propagated back to the feature extracting models to iteratively improve the learned feature representations.

Figure 2 details the 7 different fusion model architectures that we experimented in this study. Each input modality is color coded. Our **Early Fusion** model is a simple fully connected neural network model, taking in a concatenation of all the EMR features as well as the learned feature representation from the last fully connected layer of the PENet. In total, we implemented 4 different types of late fusion models. **Late NN Average Fusion** model takes the average of the predicted probabilities from the PENet model and a Neural Network trained simultaneously with all the EMR features. **Late Elastic Average Fusion** uses an ElasticNet instead of a feed forward neural network for the EMR features. **Late Separate Average Fusion** takes an average of the predicted probabilities of 7 different neural networks for each type of EMR data (including PENet). **Late Meta Fusion** uses a meta neural network classifier trained with the predicted probabilities from each of the 7 single modality classifiers. Lastly, our two joint fusion models, **Joint All Fusion** and **Joint Separate Fusion** differ by whether different EMR features are passed into a single feature extraction neural network or separately neural networks.

For all feed-forward neural network models (Fusion and EMR only), we utilized a grid search approach to find the optimal activation [ELU, LeakyReLU, Tanh], number of hidden layers [0–10], number of neurons [10–500], optimizer [Adam, SGD, AdaDelta], learning rate [0.0001–0.1], weight initialization method [Normal, Xavier, Kaiming], and dropout rate [0.3–0.8]. All of the models are trained with a batch size of 256 and a total of 200 epochs. The optimal weights for each model are saved based on the epoch that achieved the highest validation accuracy. The best model is also chosen based on the configuration that gives the lowest validation loss.

### Statistical analysis

Area under the receiver operating characteristic curve (AUROC) for each of the fusion models was used to determine the best performing model. To comprehensively compare the performance of the best fusion model to the single modality models, several evaluation metrics were calculated for the performance across the entire test set, including: AUROC, sensitivity, specificity, accuracy, positive predictive value (PPV), and negative predictive value (NPV). Diagnosing subsegmental-only PE is known to have questionable clinical significance and is often left untreated^36^. Therefore, we have also computed the same evaluation metrics for negative and non-subsegmental-only positive PE to understand the clinical utility of our model. Lastly, we calculated 95% DeLong Confidence Intervals for the AUROC of the model, and 95% Wilson Score Confidence Intervals for sensitivity, specificity, accuracy, PPV, and NPV at each operating point to measure the variability in these estimates. All confidence intervals were calculated with 1000 empirical bootstrap replicates.

### Relevant guidelines

All applicable institutional IRB guidelines were followed as well as relevant state and national data privacy regulations.

### Informed consent

This study was approved by the IRB of Stanford University and patient consent was waived by the same.

## Supplementary Information

Supplementary Information.

## Data Availability

The datasets generated and analyzed during the study are not currently publicly available due to HIPAA compliance agreement but are available from the corresponding author on reasonable request.

## Code availability

All code related to this project will be made available at https://github.com/marshuang80/pe_fusion.
