# Supplementary material for: Multimodal fusion with deep neural networks for leveraging CT imaging and electronic health record: a case-study in pulmonary embolism detection
Source: Sci Rep. 2020 Dec 17;10:22147. doi: 10.1038/s41598-020-78888-w (PMC7746687; doi:10.1038/s41598-020-78888-w)
Supplement: Supplementary file 1 — Supplementary Information. [file 41598_2020_78888_MOESM1_ESM.pdf]

Multimodal Fusion with Deep Neural Networks for Leveraging CT Imaging and Electronic Health Record - A Case-study in Pulmonary Embolism Detection (Supplementary)

Shih-Cheng Huang[*,1,2], Anuj Pareek[2,3], Roham Zamanian[4,5], Imon Banerjee[2,6], Matthew P Lungren[1,2,3]

[1] Department of Biomedical Data Science, Stanford University

[2] Center for Artificial Intelligence in Medicine & Imaging, Stanford University

[3] Department of Radiology, Stanford University

[4] Department of Pulmonary Critical Care Medicine, Stanford University

[5]Vera Moulton Wall Center for Pulmonary Vascular Disease, Stanford University School of Medicine, Stanford University

[6] Department of Biomedical Informatics, Emory University

*Corresponding Author

email: {mschuang}@stanford.edu

Supplementary Material

Model Type	Train Acc	Val Acc	Loss	Dropout prob	Weight Init	Learning rate	Optimizer	Num Layers	Num Neuron	Activation
**Demo**	58.29%	54.92%	0.6756	0.8	Kaiming	0.05	SGD	3	500	ELU
**Labs**	60.89%	66.84%	0.6078	0.8	Normal	0.1	SGD	2	200	ELU
**Vitals**	57.86%	59.07%	0.6695	0.3	Xavier	0.005	Adam	5	500	Tanh
**INP_MED**	55.87%	62.18%	0.6500	0.8	Xavier	0.005	Adam	5	200	ELU
**OUT_MED**	55.74%	61.66%	0.6691	0.5	Normal	0.005	Adam	2	100	Tanh
**ICD**	91.40%	89.12%	0.3752	0.5	Kaiming	0.005	Adam	5	10	Tanh
**All**	92.83%	81.87%	0.4168	0.3	Xavier	0.05	Adadelta	1	10	Tanh
**Joint All**	86.73%	81.34%	0.5202	0.8	Xavier	0.01	Adam	3	100	Tanh
**Joint Separate**	95.59%	81.35%	0.4815	0.8	Kaiming	0.001	Adam	3	100	LeakyRelu
**Early**	96.67%	72.44%	0.1771	0.3	Xavier	0.1	Adadelta	1	200	Tanh
**Late Meta**	92.63%	89.11%	0.3279	0.5	Xavier	0.01	Adam	5	100	LeakyRelu

Supplementary Table S1: Parameters of the best performance models using grid search. For all feed-forward neural network models (Fusion and EMR only), we utilized a grid search approach to find the optimal activation [ELU, LeakyReLU, Tanh], number of hidden layers [0-10], number of neurons [10-500], optimizer [Adam, SGD, AdaDelta], learning rate [0.0001-0.1], weight initialization method [Normal, Xavier, Kaiming], and dropout rate [0.3-0.8]. All of the models are trained with a batch size of 256 and a total of 200 epochs. The optimal weights for each model are saved based on the epoch that achieved the highest validation accuracy. The best model is also chosen based on the configuration that gives the lowest validation loss.

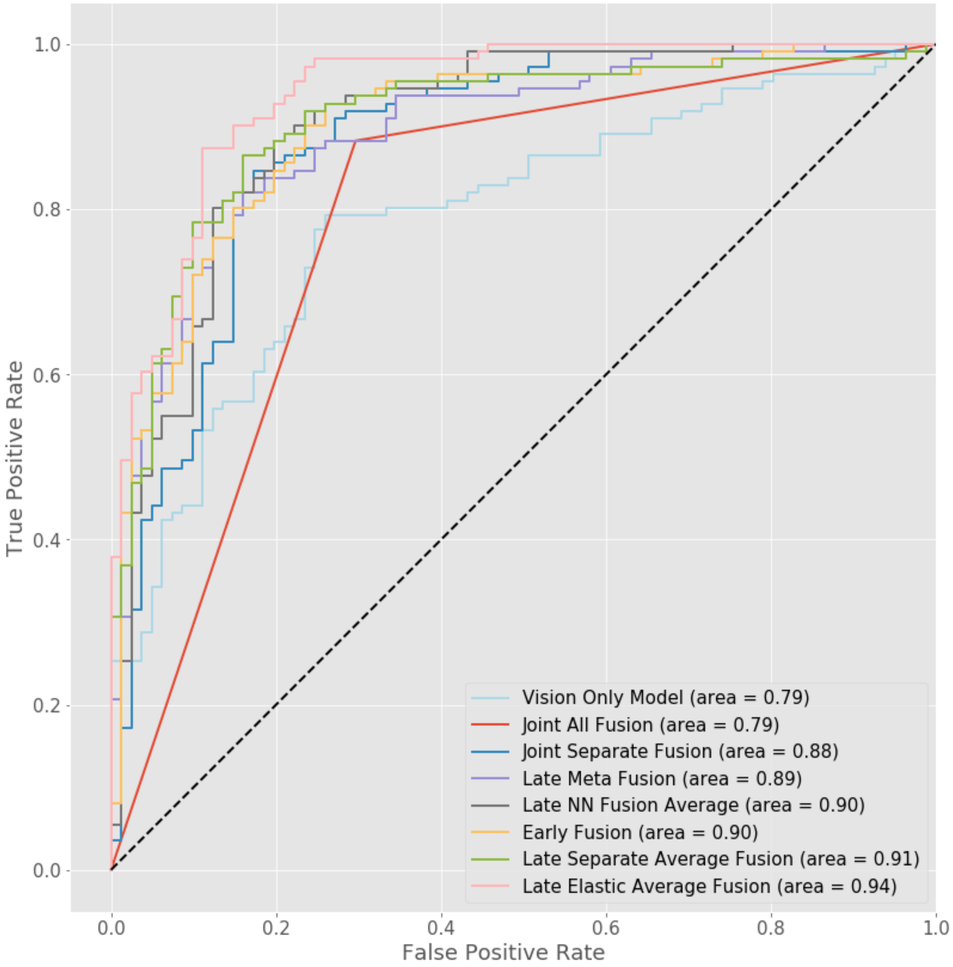

Supplementary Figure S2: **Comparison of performance under different fusion strategies.** The Receiver operating characteristic curve (ROC) for the 7 different fusion models and the vision only model (PENet). Late Elastic Average Fusion achieved the highest AUROC of 0.94, which is 0.15 AUROC higher than the single modality vision only model.

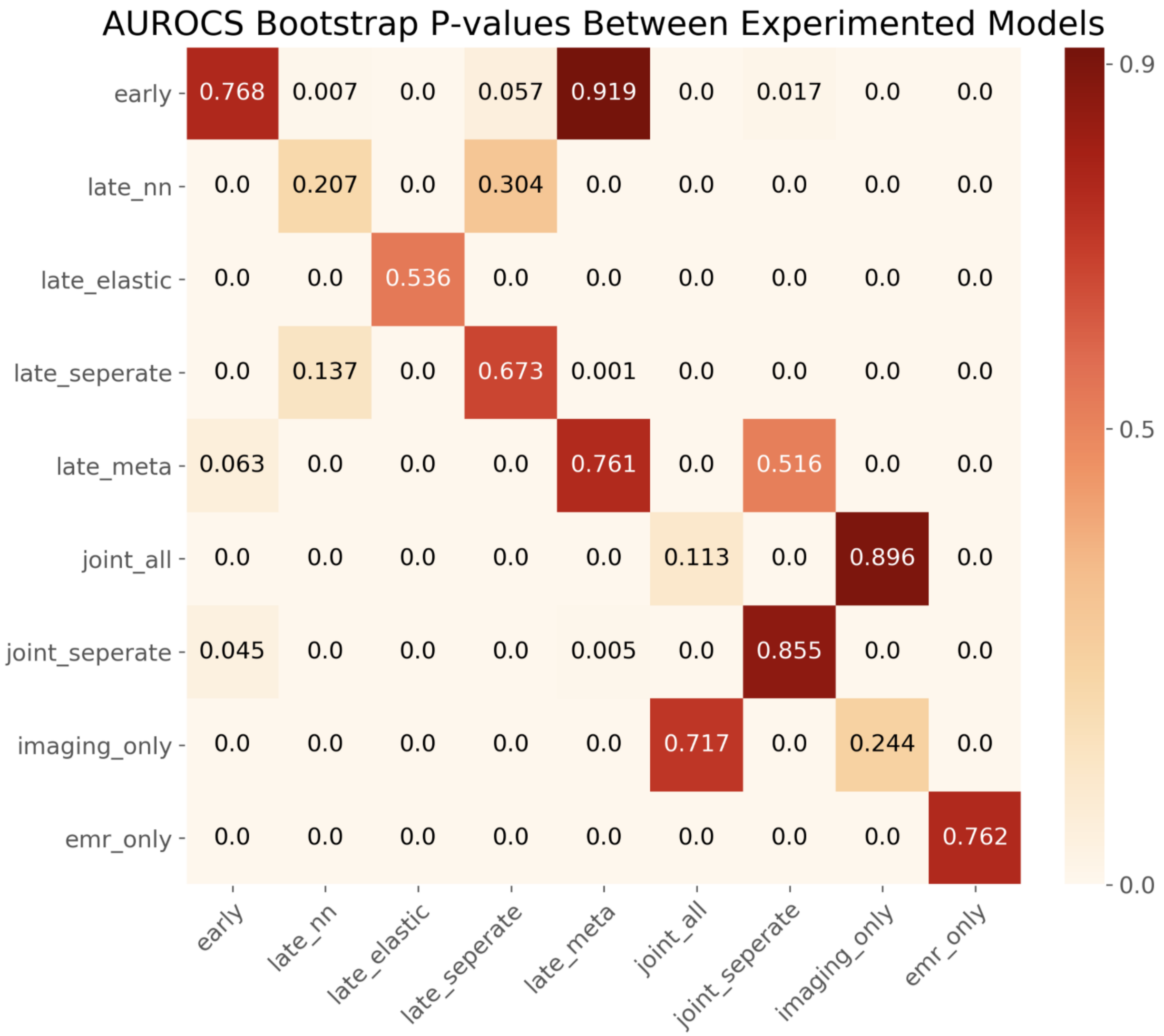

Supplementary Figure S3: AUROC Bootstrap P-values between experimented models. For each pair of models, bootstrap AUROCs were calculated by randomly sampling a subset of each model's predicted probability for 100 times, and the two-sample t-test was used to determine if there was a statistically significant difference between them. We consider two model's performance to be significantly different if $p < 0.0011$ (multiple hypothesis correction).

**Ground Truth**	**EMR Prediction**	**Image Prediction**	**Pre-Existing Medical Conditions on CT-scan**
0	1	0	Patient has aortic aneurysm and hematuria. Hazy appearance in CT may be related to pulmonary edema. Thrombus in the left popliteal and superficial femoralveins.
0	1	0	Patient has breast cancer - cancer increase risk for PE
0	1	1	Consolidation of the lung bases which may be due to atelectasis versus airspace disease

0	1	0	History of prothrombin mutation and pulmonary embolism. Previously identified with subsegmental PE. Presented with dyspnea and tachycardia. Increased size of right ventricles, need correlation with echocardiogra.
0	1	1	Azygous lobe present. Ground-glass opacity in the middle lobe. Calcified granuloma in right lower lobe. Recommend abdomen and pelvis CT scans and clinical correlation.
0	1	0	Right upper and lower lobe consolidation. Bibasilar atelectasis.
0	1	1	Thorax is highly deformed because of scoliosis
0	1	1	Patient has moderate pleural effusion
0	1	1	Patient has multifocal scarring and/or atelectasis is scattered about the lungs

Supplementary Table S4: Analysis of the false positive predictions made by the fusion model. Examining radiologist's notes on the false positive cases indicated that all of these studies had pre-existing or concurrent medical conditions.

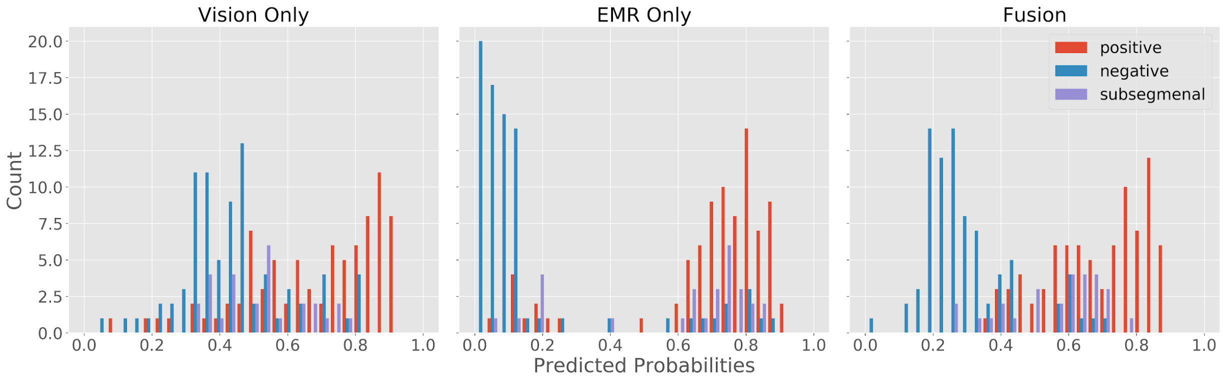

Supplementary Figure S5: **Analysis of predicted probabilities.** Predicted probability across all positive, negative and subsegmental cases on the held-out test set using (left) Vision only model, (mid) EMR only model and (right) Fusion model. Vision only model showed large overlapping regions of predicted probabilities for the positive and negative test cases. The EMR only model revealed more defined clusters of the same cases but still suffered from limited separation. The multimodal fusion model achieves more clinically useful separation between positive and negative cases; all the central and segmental positive cases can be diagnosed correctly with an operating point of 0.35 and achieving a specificity 0.778.
